# *Hypericum japonicum* Thunb. ex Murray: Phytochemistry, Pharmacology, Quality Control and Pharmacokinetics of an Important Herbal Medicine

**DOI:** 10.3390/molecules190810733

**Published:** 2014-07-24

**Authors:** Lin-Sheng Liu, Meng-Hua Liu, Jing-Yu He

**Affiliations:** 1 Department of Pharmacy, Foshan Hospital of Traditional Chinese Medicine, Foshan 528000, Guangdong, China; E-Mail: llsgqy@163.com; 2 School of Pharmaceutical Sciences, Southern Medical University, Guangzhou 510515, Guangdong, China; E-Mail: menghua_liu@hotmail.com; 3 Guangzhou Institute of Advanced Technology, Chinese Academy of Sciences, Guangzhou 511458, Guangdong, China

**Keywords:** *Hypericum japonicum*, phytochemistry, pharmacology, quality control, pharmacokinetics

## Abstract

*Hypericum japonicum* Thunb. ex Murray is mainly distributed throughout Asia, Oceania and North America and is used as an important herbal medicine. *H. japonicum* contains many valuable secondary metabolites, such as flavonoids, phloroglucinols and xanthones and has hepatoprotective, anti-tumor, antibacterial, antiviral, and antioxidant activities and effects on the cardiovascular system and immunity. Coupled with phytochemical and pharmacological research, a series of analytical methods have been developed to evaluate the quality of *H. japonicum* based on its bioactive components. A pharmacokinetics study involved the absorption of two main flavonoids of *H. japonicum* in rats. This review aims to present an up-to-date and comprehensive overview of the phytochemistry, pharmacology, quality control and pharmacokinetics of *H. japonicum*, which should be useful for the greater development of *H. japonicum*, especially in the development of new drugs and therapeutics for various diseases.

## 1. Introduction

*Hypericum japonicum* Thunb. ex Murray (Hypericaceae) is an annual herb mainly distributed throughout Asia, Oceania and North America [1,2]. In China, it is widely distributed in Liaoning Province, Shandong Province and other provinces in southern China where it grows in rice fields, ditches, marshes, grasslands and waste places where the altitude is below 2800 m [2,3].

*H. japonicum* called “Tian-Ji-Huang” or “Di-Er-Cao” in Chinese was first listed as a herbal medicine in “Sheng Cao Yao Xing Bei Yao” published during the Qi Dynasty. It has been used in Traditional Chinese Medicine for a long time for relieving internal heat or fever, hemostasis and detumescence [4]. In addition, it has also been used as a medicinal herb in Asian countries, such as Japan, South Korea, Thailand, Nepal, India, Vietnam and Philippines [2]. *H. japonicum* has been studied due to its high value in traditional medicine. The results of modern pharmacological studies have shown that *H. japonicum* could be used for the treatment of bacterial diseases, infectious hepatitis, acute and chronic hepatitis, gastrointestinal disorders, internal hemorrhages and tumors, which generally matches its traditional uses [4,5]. So far, no studies on the toxicity of *H. japonicum* have been reported.

Recently, different classes of chemicals such as flavonoids, xanthonoids, chromone glycosides, phloroglucinol derivatives and lactones have been found in this plant. Among these isolated components, some single flavonoid compounds such as quercitrin, isoquercitrin and quercetin-7-O-α-L-rhamnose are shown to have a variety of bioactivities *in vivo* or *in vitro*, and thereby are thought as the bioactive components of *H. japonicum*. Hence, quality control based on these bioactive components to ensure the effects of *H. japonicum* materials and its related products is urgent and necessary. However, the quality control of *H. japonicum* is still not listed in the Chinese Pharmacopoeia (2010 edition) and other official pharmacopoeias. A number of studies have thus attempted to develop accurate, sensitive and selective analytical methods for qualitative and quantitative evaluation of *H. japonicum*.

Aiming to provide beneficial information for modern uses and scientific studies of *H. japonicum*, this review summarizes and evaluates the available phytochemical and bioactive properties of *H. japonicum* reported by the literature. Besides, the research progress in the quality evaluation and pharmacokinetics of *H. japonicum* are also presented.

## 2. Phytochemistry

**Figure 1:**
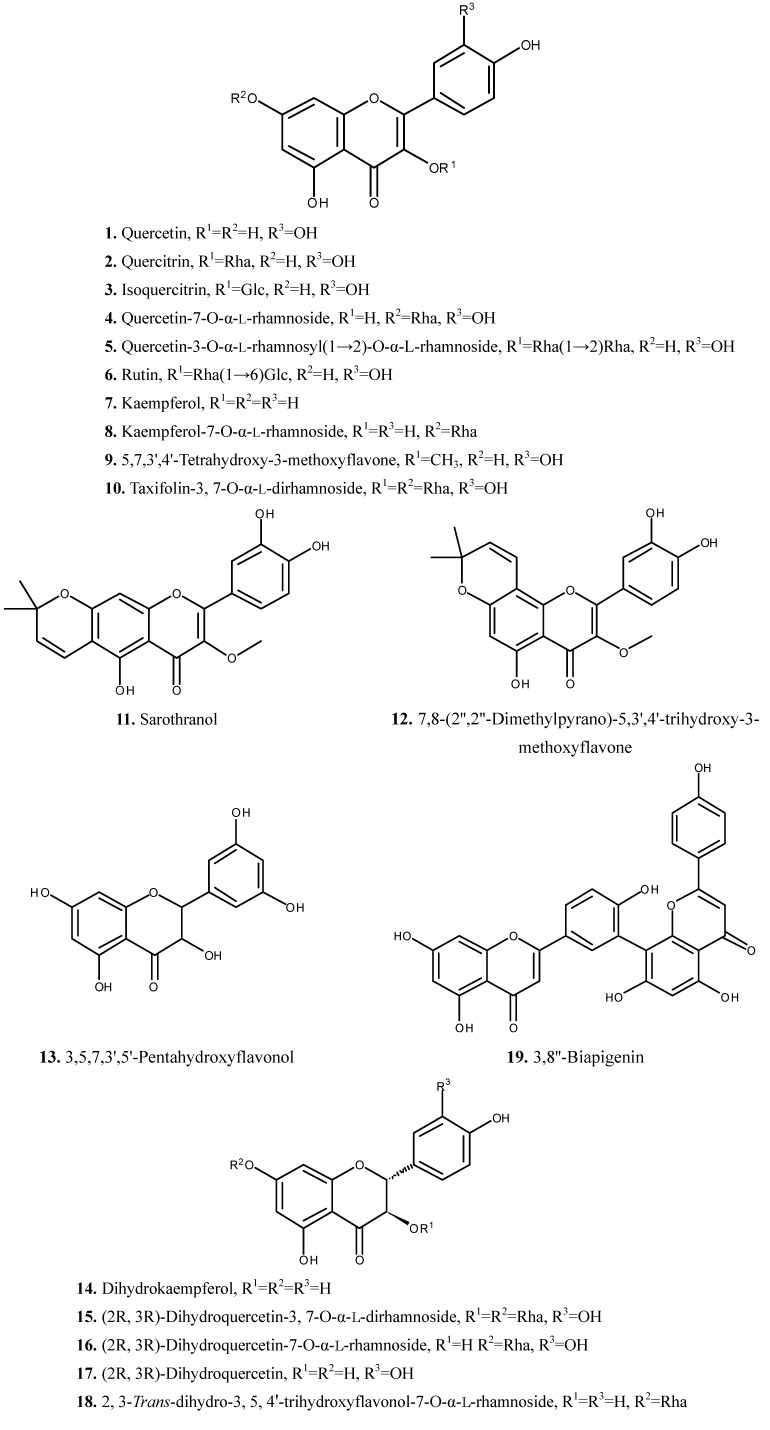
Chemical structures of flavonoids **1**–**19** from *H. japonicum*.

**Figure 2:**
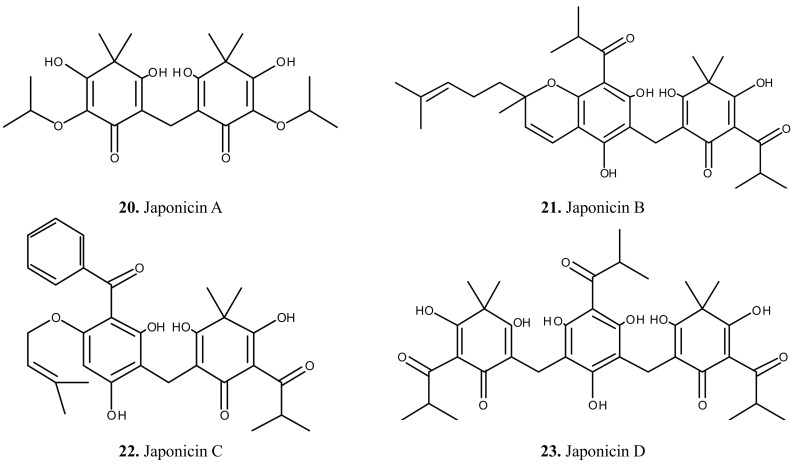

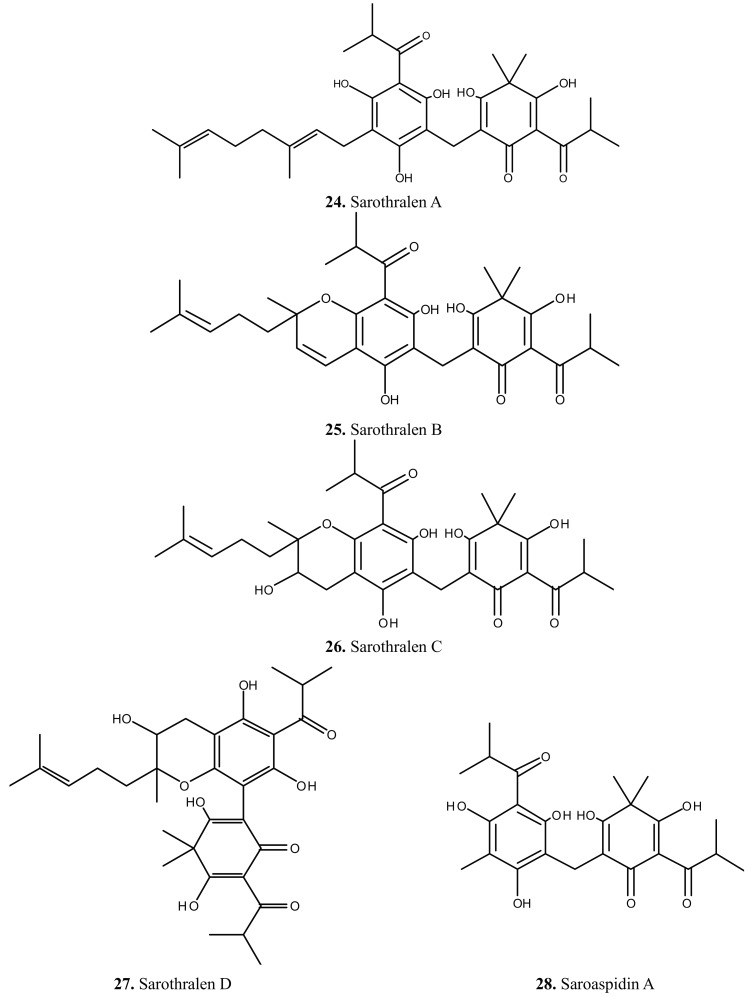

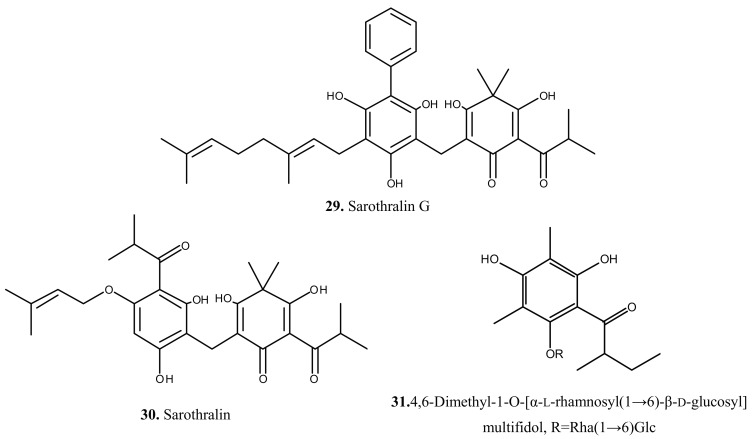
Chemical structures of phloroglucinols **20**–**31** from *H. japonicum*.

**Figure 3:**
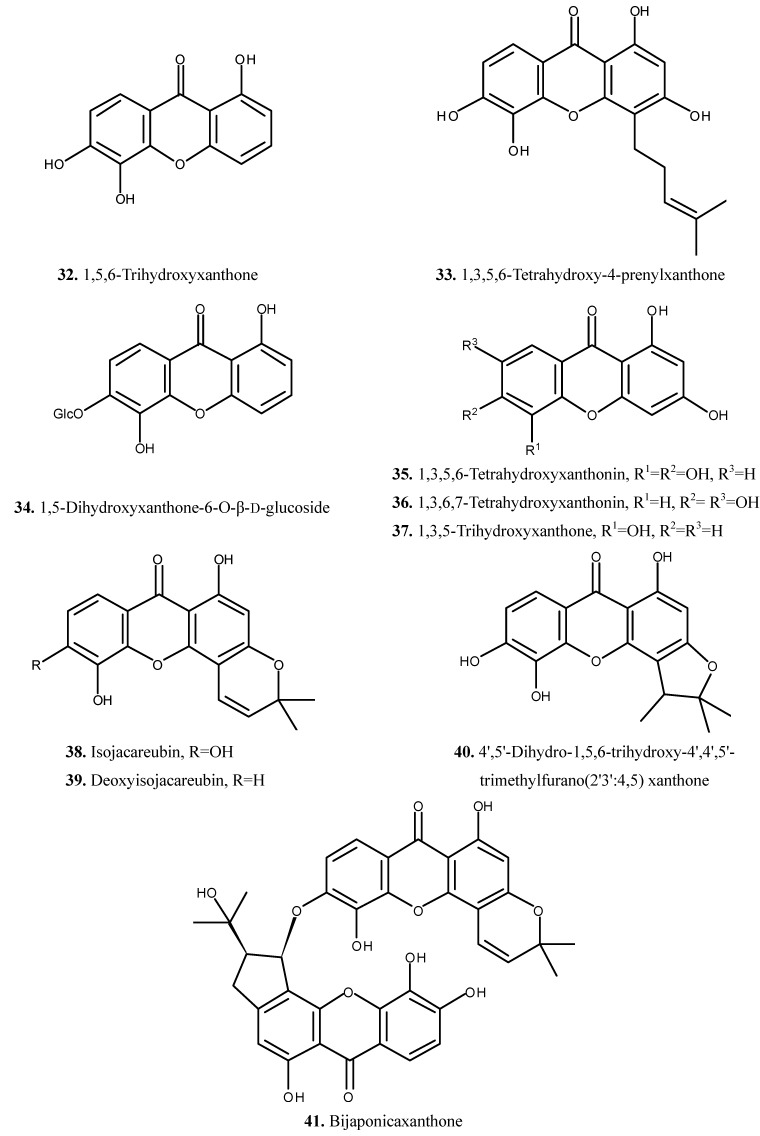
Chemical structures of xanthones (**32**–**41**) from *H. japonicum*.

**Figure 4:**
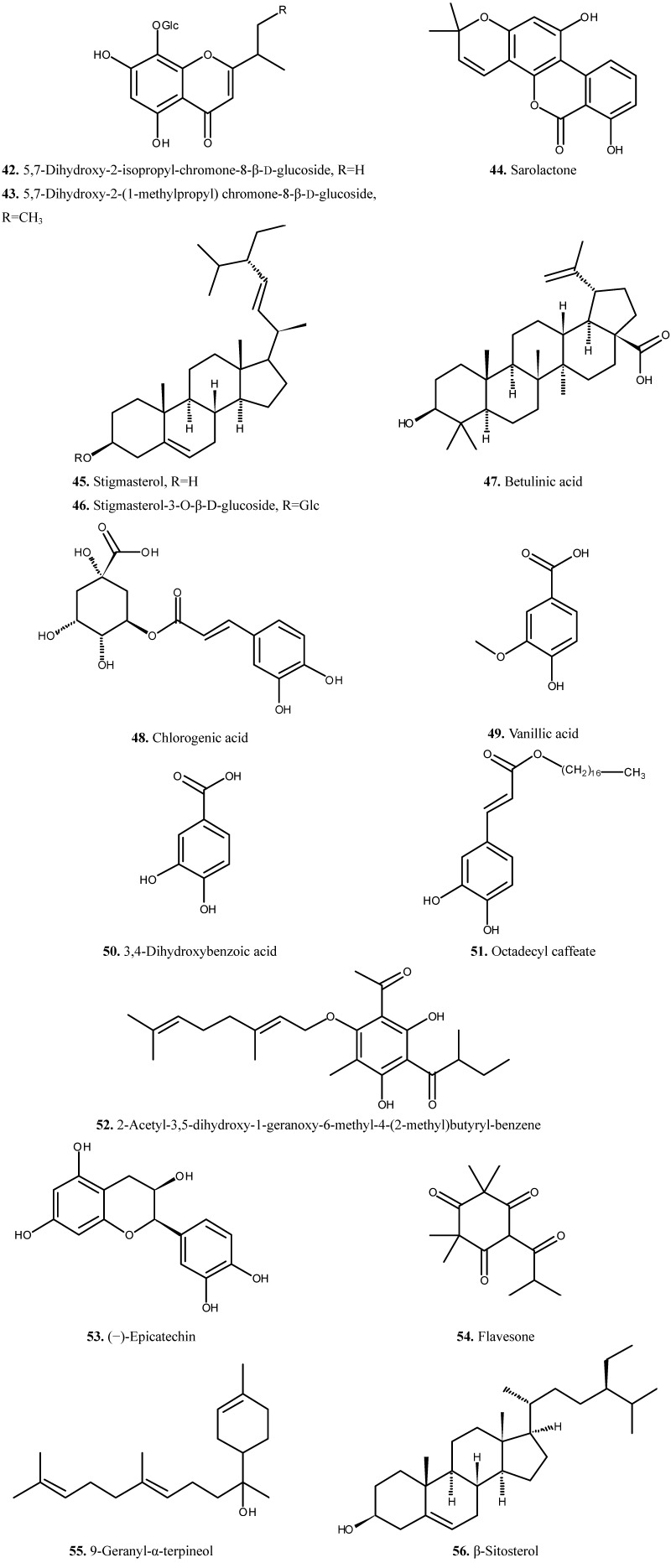
Chemical structures of other compounds (**42**–**56**) from *H. japonicum*.

The chemical composition of *H. japonicum* has been studied during the last few years due to the importance and availability of plant. The phytochemical studies on *H. japonicum* have resulted in the isolation of flavonoids, phloroglucinols and xanthones [6,7,8,9,10,11,12,13,14,15,16,17,18,19,20]. In addition, some compounds from other classes were also isolated from this species [6,7,21,22,23,24]. The isolated compounds (compounds **1**–**56**) are summarized in Table 1 and their chemical structures are presented in Figure 1, Figure 2, Figure 3 and Figure 4.

**Table 1:** Compounds in *H. japonicum*.

No.	Compounds	Classes	References	
**1**	Quercetin	Flavonoid	[6]	
**2**	Quercitrin	Flavonoid	[6]	
**3**	Isoquercitrin	Flavonoid	[7]	
**4**	Quercetin-7-O-α-l-rhamnoside	Flavonoid	[7]	
**5**	Quercetin-3-O-α-l-rhamnosyl(1→2)-O-α-l-rhamnoside	Flavonoid	[7]	
**6**	Rutin	Flavonoid	[8]	
**7**	Kaempferol	Flavonoid	[7]	
**8**	Kaempferol-7-O-α-l-rhamnoside	Flavonoid	[7]	
**9**	5,7,3',4'-Tetrahydroxy-3-methoxyflavone	Flavonoid	[9]	
**10**	Taxifolin-3,7-O-α-l-dirhamnoside	Flavonoid	[10]	
**11**	Sarothranol	Flavonoid	[11]	
**12**	7,8-(2'',2''-Dimethylpyrano)-5,3',4'-trihydroxy-3-methoxyflavone	Flavonoid	[7]	
**13**	3,5,7,3',5'-Pentahydroxyflavonol	Flavonoid	[9]	
**14**	Dihydrokaempferol	Flavonoid	[12]	
**15**	(2R,3R)-Dihydroquercetin-3,7-O-α-l-dirhamnoside	Flavonoid	[7]	
**16**	(2R,3R)-Dihydroquercetin-7-O-α-l-rhamnoside	Flavonoid	[7]	
**17**	(2R,3R)-Dihydroquercetin	Flavonoid	[7]	
**18**	2,3- *Trans*-dihydro-3,5,4'-trihydroxyflavonol-7-O-α-l-rhamnoside	Flavonoid	[7]	
**19**	3,8''-Biapigenin	Flavonoid	[6]	
**20**	Japonicin A	Phloroglucinol	[13]	
**21**	Japonicin B	Phloroglucinol	[13]	
**22**	Japonicin C	Phloroglucinol	[13]	
**23**	Japonicin D	Phloroglucinol	[13]	
**24**	Sarothralen A	Phloroglucinol	[14]	
**25**	Sarothralen B	Phloroglucinol	[14]	
**26**	Sarothralen C	Phloroglucinol	[15]	
**27**	Sarothralen D	Phloroglucinol	[15]	
**28**	Saroaspidin A	Phloroglucinol	[16]	
**29**	Sarothralin G	Phloroglucinol	[17]	
**30**	Sarothralin	Phloroglucinol	[18]	
**31**	4,6-Dimethyl-1-O-[α-l-rhamnosyl(1→6)-β-d-glucosyl] multifidol	Phloroglucinol	[19]	
**32**	1,5,6-Trihydroxyxanthone	Xanthone	[7]	
**33**	1,3,5,6-Tetrahydroxy-4-prenylxanthone	Xanthone	[7]	
**34**	1,5-Dihydroxyxanthone-6-O-β-d-glucoside	Xanthone	[7]	
**35**	1,3,5,6-Tetrahydroxyxanthonin	Xanthone	[20]	
**36**	1,3,6,7-Tetrahydroxyxanthonin	Xanthone	[20]	
**37**	1,3,5-Trihydroxyxanthone	Xanthone	[20]	
**38**	Isojacareubin	Xanthone	[7]	
**39**	Deoxyisojacareubin	Xanthone	[7]	
**40**	4',5'-Dihydro-1,5,6-trihydroxy-4',4',5'-trimethylfurano(2'3':4,5) xanthone	Xanthone	[7]	
**41**	Bijaponicaxanthone	Xanthone	[7]	
**42**	5,7-Dihydroxy-2-isopropyl-chromone-8-β-d-glucoside	Chromone	[7]	
**43**	5,7-Dihydroxy-2-(1-methylpropyl) chromone-8-β-d-glucoside	Chromone	[7]	
**44**	Sarolactone	Chromone	[21]	
**45**	Stigmasterol	Triterpene	[6]	
**46**	Stigmasterol-3-O-β-d-glucoside	Triterpene	[6]	
**47**	Betulinic acid	Triterpene	[6]	
**48**	Chlorogenic acid	Phenolic acid	[12]	
**49**	Vanillic acid	Phenolic acid	[22]	
**50**	3,4-Dihydroxybenzoic acid	Phenolic acid	[23]	
**51**	Octadecyl caffeate	Phenol	[23]	
**52**	2-Acetyl-3,5-dihydroxy-1-geranoxy-6-methyl-4-(2-methyl)butyryl-benzene	Phenol	[24]	
**53**	(−)-Epicatechin	Phenol	[23]	
**54**	Flavesone	Ketone	[24]	
**55**	9-Geranyl-α-terpineol	Alcohol	[24]	
**56**	β-Sitosterol	Sterol	[23]	

### 2.1. Flavonoids

Flavonoids are very common and important secondary metabolites in Nature. So far, nineteen flavonoids have been found in *H. japonicum*, including quercetin (**1**), quercitrin (**2**), isoquercitrin (**3**), quercetin-7-O-α-l-rhamnoside (**4**), quercetin-3-O-α-l-rhamnosyl(1→2)-O-α-l-rhamnoside (**5**), rutin (**6**), kaempferol (**7**), kaempferol-7-O-α-l-rhamnoside (**8**), 5,7,3',4'-tetrahydroxy-3-methoxyflavone (**9**), taxifolin-3,7-O-α-l-dirhamnoside (**10**), sarothranol (**11**), 7,8-(2'',2''-dimethylpyrano)-5,3',4'-trihydroxy-3-methoxyflavone (**12**), 3,5,7,3',5'-pentahydroxyflavonol (**13**), dihydrokaempferol (**14**), (2*R*,3*R*)-dihydroquercetin-3,7-O-α-l-dirhamnoside (**15**), (2*R*,3*R*)-dihydroquercetin-7-O-α-l-rhamnoside (**16**), (2*R*,3*R*)-dihydroquercetin (**17**), 2,3-*trans*-dihydro-3,5,4'-trihydroxyflavonol-7-O-α-l-rhamnoside (**18**) and 3,8''-biapigenin (**19**) (Figure 1).

### 2.2. Phloroglucinols

Phloroglucinol derivatives were the other main components of *H. japonicum*. Eleven compounds, including japonicins A–D (**20**–**23**), sarothralens A–D (**24**–**27**), saroaspidin A (**28**), sarothralin G (**29**) and sarothralin (**30**) were isolated from this species two decades ago. After that, only one new phloroglucinol named 4,6-dimethyl-1-O-[α-l-rhamnosyl(1→6)-β-d-glucosyl] multifidol (**31**) was obtained from this species (Figure 2).

### 2.3. Xanthones

The xanthone derivatives, 1,5,6-trihydroxyxanthone (**32**), 1,3,5,6-tetrahydroxy-4-prenylxanthone (**33**), 1,5-dihydroxyxanthone-6-O-β-d-glucoside (**34**), 1,3,5,6-tetrahydroxyxanthonin (**35**), 1,3,6,7-tetra-hydroxyxanthonin (**36**), 1,3,5-trihydroxyxanthone (**37**), isojacareubin (**38**), deoxyisojacareubin (**39**), 4',5'-dihydro-1,5,6-trihydroxy-4',4',5'-trimethylfurano(2',3':4,5) xanthone (**40**) and bijaponicaxanthone (**41**) have been isolated and identified (Figure 3).

### 2.4. Other Compounds

Three chromones **42**–**44**, three triterpenes **45**–**47**, three phenolic acids **48**–**50**, three phenols **51**–**53**, a ketone **54**, an alcohol **55** and a sterol **56** have been obtained during the phytochemical studies (Figure 4).

**Table 2:** The main volatile components of *H. japonicum*.

Roots		Aerial Parts	
Compound	Relative Percentage	Compound	Relative Percentage
Dodecyl acetate	20.59%	Undecane	19.25%
Decyl dichlorocetate	13.09%	Dodecyl acetate	16.86%
3-Methyl oxirane-2-methanol	9.37%	(*E*)-β-Farnesene	10.84%
Capraldehyde	8.41%	β-Curcumin	10.32%
β-Caryophyllene	8.13%	Tetradecanol	6.54%
(*E*)-β-Farnesene	5.74%	2,6-Bimethyl-6-(4-methyl-3-pentenyl)bicyclo[3.1.1]hept-2-ene	6.15%
Nonane	5.18%		

### 2.5. Volatile Oil

The volatile oil extracted from the roots and the aerial parts (stems and leaves) of *H. japonicum* were analyzed by GC-MS, and thirty-two and forty-three constituents were identified, respectively. As the results show, there was obvious chemical variability in the volatile oil composition: the contents of six chemotyppes including hydrocarbons, alcohols (phenols), aldehydes/ketones, acids, esters and amines were 31.92%, 11.47%, 9.95%, 0.56%, 40.03% and 4.13% in the roots, respectively, and 62.16%, 8.12%, 2.72%, 1.24%, 18.96% and 5.75% in the aerial parts, respectively [25]. The main volatile components that represented more than 5% of the total volatile oils are summarized in Table 2.

### 2.6. Metallic Elements

Fifteen rare earth elements in wild *H. japonicum* materials were analyzed by inductively coupled plasma mass spectrometry (ICP-MS). The results showed that the concentrations of La, Ce, Pr, Nd, Sm, Eu, Gd, Tb, Dy, Ho, Er, Yb, Tm, Lu and Y ranged from 6 to 14522 ng/g, and among them the concentrations of La, Ce and Nd were higher than 2,000 ng/g [26]. In addition, the contents of other five metallic elements, Zn, Cu, Pb, Cr and Cd were measured by flame atomic absorption spectrophotmetry (FAAS) [27].

## 3. Pharmacology

### 3.1. Antioxidant Activity

The antioxidant effect of *H. japonicum* is one of the most prominent effects due to its responsibility for many of the other activities. The aqueous extracts of *H. japonicum* have been demonstrated to have obvious antioxidant activity by molybdenum reduction, DPPH scavenging, β-carotene bleaching inhibition and lipid peroxidation inhibition methods with values of 37.28 ± 0.54 μg/mg, IC_50_ = 77.7 ± 5.6 μg, 83.18% and 95.38%, respectively. By reducing the generation of hydroxyl radicals, the aqueous extract effectively reduced the oxidative damage of the DNA [28].

Quercetin-7-O-α-l-rhamnoside is one of main flavonoids in *H. japonicum*. Oral treatment with quercetin-7-O-α-l-rhamnoside (0.5, 1.0 and 2.0 mg/kg) in bile duct ligation-injured liver fibrosis rats showed increases of superoxide dismutase (SOD) and glutathione peroxidase (GSH-Px) level, and a decrease of malondialdehyde (MDA) content in liver. *In vitro*, inhibiting the overexpression of ROS and GSH depletion is a very important reason for quercetin-7-O-α-l-rhamnoside to attenuate L-02 cell injury induced by glycochenodeoxycholic acid [29].

### 3.2. Hepatoprotective Activity

Total bilirubin (TBIL), alanine transaminase (ALT) and aspartate transaminase (AST) in serum are three markers for liver function. After intraperitoneal administration with ethanol and ethyl acetate extract of *H. japonicum* (0.2, 0.6 and 1.8 g raw materials/kg) to rats with acute liver injury induced by D-aminogalactose, the levels of ALT and AST in all treatment groups were reduced remarkably when compared to those in the model group [30]. The aqueous extract (4.5 g raw material/kg) exhibited an obvious effect by decreasing AST, ALT and TBIL levels in serum of mice with liver injury induced by CCl_4_, indicating its hepatoprotective effects [31]. When three flavonoids isolated from *H. japonicum*, namely quercitrin, isoquercitrin and quercetin-7-O-α-l-rhamnoside (0.25, 0.5 and 1.0 mg/kg), were used separately to treat the liver injury in rats induced by CCl_4_ and D-aminogalactose, the levels of AST, ALT and TBIL were significantly reduced at the three dose levels as compared to the model group [32]. Furthermore, intraperitoneal treatment with quercetin-7-O-α-L-rhamnoside (0.25, 0.5 and 1.0 mg/kg) of rats with liver fibrosis induced by bile duct ligation showed a reduction in levels of type III procollagen, hyaluronic acid, laminin and TNF-α in serum and expression of smooth muscle actin-α in liver [33].

### 3.3. Anti-Cancerous Activity

The anti-cancerous effect is a noticeable bioactivity for *H. japonicum* reported in the last decade. *H. japonicum* extract significantly inhibited the proliferation of human tongue cancer cell line TSCCa *in vitro* in a dose-dependent manner by damaging the mitochondria and rough endoplasmic reticulum [34]. The extract of *H. japonicum* significantly inhibited the proliferation of CNE-2 cells and HepG2 cells *in vitro* by inducing cell cycle arrest, showing a dose-dependent response at the low concentrations of 25 mg/mL and 5 mg/mL, respectively [35,36,37]. Meanwhile, the aqueous extract of *H. japonicum* displayed a synergistic tumor-inhibiting effect with 5-FU in mice at 3, 6 and 12 g/kg/day, administered 24 h after the tumor inoculation, once daily for 10 days, indicting its usefulness in antitumor therapy [38]. Using a serum pharmacology method, BEL-7404 liver cancer cells were co-cultured with the collected serum containing four fraction of *H. japonicum* (ethanol extract, ethyl acetate extract, *n*-butanol extract and aqueous extract) at different dosage. All extracts showed inhibitory activity on the growth of BEL-7404 cell with inhibitory rates of 29.74%, 53.80%, 40.79% and 54.24%, respectively [39].

### 3.4. Antibacterial Activity

Antibacterial activity for aqueous extract of *H. japonicum* was studied by the disc diffusion method. The results demonstrated that both Gram positive and Gram negative bacteria, including *Escherichia coli*, *Alcaligens faecalis*, *Bacillus subtilis*, *E. aerogenes*, *Klebsiella pneumonia*, *Shigella flexneri*, *Salmonella enterica* ser. Typhi, *Staphylococcus aureus*, *Staphylococcus epidermidis*, *Streptococcus pyogenes*, *Xanthomonas vesicatoria*, *X. oryzae* pv. oryzae and *X. malvacearum* were inhibited by the extract, except for *P. aeruginosa*, and the minimum inhibitory concentration (MICs) of the extract was 1 mg/mL against all the test cultures used, except *E. aerogenes* and *P. aeruginosa*. This indicated the extract had a broad spectrum antibacterial activity [28].

Isojacareubin, the xanthone from the aerial parts of *H. japonicum* had an effect on methicillin-resistant *Staphylococcus aureus* (MRSA) with MICs/MBCs ranging from 4/16 to 16/64 μg/mL. When it was used together with some conventional antibacterial agents, namely ceftazidime, levofloxacin and ampicillin, the values of 50% of the fractional inhibitory concentration indices (FICI_50_) were 0.25, 0.37 and 0.37, respectively, indicating good anti-MRSA activities [40].

### 3.5. Antiviral Activity

Using a serum pharmacology method, the anti-HBV effect for different extracts (ethanol extract, ethyl acetate extract, *n*-butanol extract and aqueous extract) of *H. japonicum* were tested *in vitro*. The aqueous extract showed better anti-HBV activity than the other three extracts with inhibitory rates on HBeAg and HBsAg of 70% and 30%, respectively [39]. In an *in vivo* study, *H. japonicum* extract showed strong activity against duck hepatitis B virus at dosage of 6.5, 13.0 and 26.0 mg/kg, once a day, for 28 days [41]. In addition, the 75% ethanol extract of *H. japonicum* exhibited anti-influenza virus H3N2 effect *in vivo* after 10 g/kg oral administration in mice infected with the H3N2 virus [42].

### 3.6. Effects on Cardiovascular System

The bioactive compound quercetin-7-O-α-L-rhamnoside isolated from *H. japonicum* was tested for its coagulant activity via an *in vitro* experiment. This flavonol glycoside (10^−5^ g/L) exhibited excellent effects on promoting the coagulation of activated partial thromboplastin time and prothrombin time in dose-dependent manner [7]. Oral administration of the aqueous extract of *H. japonicum* (11.25 g raw material/kg/day) in hyperlipidemic rats for 8 weeks, significantly reduced the serum levels of triglycerides, total cholesterol, low density lipoprotein cholesterol and atherosclerotic index, but increased the level of high density lipoprotein cholesterol. Meanwhile, the hematocrit, blood and plasma viscosity were markedly decreased in this study, indicating the aqueous extract of *H. japonicum* had an adjustment effect on hemorheology [43]. Furthermore, the contents of monocyte chemoattractant protein-1, lipoprotein associated phospholipase A2 and macrophage migration inhibitory factor in serum were also obviously decreased, indicating the inhibitory effect on the atherosclerosis process [44].

### 3.7. Effects on Immunity

The effect of *H. japonicum* extract on systemic immune functions was investigated by subcutaneous injection in rats with a dosage of 2 g raw material/kg, and the results showed that *H. japonicum* extract obviously increased the phagocytic rate of neutrophils (83.5% ± 5.1%) and enhanced the T lymphocyte ratio (68.8% ± 6.5%) in the peripheral blood of male rats [45]. In addition, the extract showed strong effects on improving the immune function, reducing the toxic effect of 5-fluorouracil and prolonging the survival time in the tumor-bearing mice. Therefore, it was considered to have immunoregulatory activity [38].

## 4. Quality Control

So far, the quality evaluation of *H. japonicum* materials has been not listed in the Chinese Pharmacopoeia (2010 edition), even though it has been used as a traditional medicine for a long time. Much effort thus is made to establish a comprehensive method for ensuring the quality of *H. japonicum*. For the safety and efficacy of herbal medicines, the first step in assuring quality is correct identification [46]. Traditionally morphological and microscopic approaches have been used for *H. japonicum* identification [3]. However, these traditional methods are very limited for *Hypericum* herbs or processed products with similar macroscopic and anatomical characteristics. DNA markers have now become the popular means for the identification of plants because genetic composition is effective and unique for each individual and is less affected by age, physiological condition, environmental factors, harvest time, storage and processing conditions [47]. Internal transcribed spacer sequence (ITS) of nuclear ribosomal DNA (nrDNA) as a common DNA marker was applied to *H. japonicum*. A series of ITS sequences of *H. japonicum* from different research groups are reported in GenBank. However, there is still no literature on a molecular method developed for accurate identification of *H. japonicum*.

Qualitative and quantitative analysis for the quality control of *H. japonicum* has mostly focused on flavonoid components since flavonoids have been proven to be largely responsible for many of the biological activities of *H. japonicum* in pharmacological studies. Thin layer chromatography (TLC) has been applied to qualitative identification of *H. japonicum* by using the flavonoid components, isoquercitrin and quercitrin as chemical markers [48]. However, TLC quantification is thought to be relatively poor in sensitivity, resolution and reproducibility. High performance liquid chromatography (HPLC) coupled with ultraviolet spectroscopy (UV) and electrospray ionization (ESI)-mass spectrometry (MS) have become the common analytical techniques for separation and quantitation of chemical markers from complicated herbal medicine extracts. HPLC methods for the quantitation of specific chemicals in *H. japonicum* are summarized in Table 3. Isojacareubin, quercetin-7-O-α-l-rhamnoside, quercetin and quercitrin, each of which was recognized as characteristic and/or bioactive component, were used as individual chemical markers for quality evaluation of *H. japonicum* materials [49,50,51,52,53]. A lot of HPLC methods on the basis of multiple components with potential bioactivities have been developed for quantitation and presenting further useful information on this species. As the results show, the contents of flavonoids considered as the bioactive components in *H. japonicum* materials were found to vary [8,54,55,56,57,58,59,60,61]. Various reasons, such as growth environment, growth periods, harvesting season, processing method, storage condition and period might cause variation in the chemical compositions of the various samples [8,56,58,59,60].

Chemical fingerprints could show the complicated components of herbal medicines, not only the naturally occurring bioactive and characteristic components, but also their relative ratios [62]. Chemical fingerprint analysis which is recognized as a reliable means for the identification and qualification of herbal medicines, has been accepted by Food and Drug Administration (2000) [63], European Medicines Agency (2001) [64], and State Food and Drug Administration of China (2000) [65] and other authorities as a strategy for quality assessment of herbal medicines. A HPLC-diode array detector (DAD) fingerprint was applied to 56 batches of *H. japonicum* materials from six provinces in China. The results showed that the most relevant factor in secondary metabolites of *H. japonicum* was collection location, followed by harvesting time [62]. Moreover, the on-line LC-MS^n^ technique, which provides a ready method for elucidation of compounds and more information in the fingerprint by reprocessing the mass spectrometry data using tandem MS, has become a powerful means in the quality control and quantitative analysis of complicated medicinal herbs. A HPLC-PAD-ESI-MS*^n^* method was successfully developed to evaluate the quality of *H. japomicum* from different origins by establishing chromatographic fingerprints, in which 20 common peaks of 10 batches of *H. japomicum* were identified [58]. Headspsce GC-MS (HSGC-MS) was used to establish the fingerprint of the volatile components from 11 batches of *H. japonicum* materials. The HSGC-MS fingerprint with 12 common peaks was characteristic and useful for quality control of *H. japonicum* materials [66]. Additionally, a high performance capillary electrophoresis (HPCE) fingerprint based on six main peaks was also established for quality control of *H. japonicum* materials [67].

**Table 3:** HPLC applications on quantitative analysis for quality control of *H. japonicum*.

Analytes	Extraction Methods	Columns		Mobile Phase	Analytical Time	Detections	References		
Isojacareubin		Refluxing extraction with 75% (v/v) methanol aqueous solution	C18 (Diamonsil, 4.6 mm × 200 mm, 5 μm)	Acetonitrile-methanol-water-phosphoric acid (45:15:50:0.05); flow rate: 1.0 mL/min		40 min	UV 254 nm	[49]		
Isojacareubin		Ultrasonic extraction with 80% (v/v) ethanol aqueous solution	C18 (Hypersil, 4.6 mm × 250 mm, 5 μm)	Acetonitrile-0.04% phosphoric acid (47:53); flow rate: 1.0 mL/min		28 min	UV 254 nm	[50]		
Quercetin-7-O-α- l-rhamnoside		Refluxing extraction with 60% (v/v) ethanol aqueous solution	C18 (Hypersil, 4.6 mm × 250 mm, 5 μm)	Acetonitrile-0.04% phosphoric acid (77:23); flow rate: 1.0 mL/min		20 min	UV 371 nm	[51]		
Quercetin		Refluxing extraction with methanol and 25% hydrochloric acid aqueous solution (3:1)	C18 (Diamonsil, 4.6 mm × 250 mm, 5 μm)	Methanol-0.06% phosphoric acid (52:48); flow rate: 1.0 mL/min		20 min	UV 360 nm	[52]		
Quercitrin		Ultrasonic extraction with ethanol	C18 (Agilent, 4.6 mm × 250 mm, 5 μm)	Acetonitrile-0.05 mol/L potassium dihydrogenphosphate (19:81); flow rate: 1.0 mL/min		40 min	UV 256 nm	[53]		
Quercetin, quercitrin and isoquercitrin		Ultrasonic extraction with 80% (v/v) methanol aqueous solution	C18 (Hypersil, 4.6 mm × 250 mm, 5 μm)	Acetonitrile-0.02 mol/L potassium dihydrogenphosphate (14:86) with gradient elution; flow rate: 1.0 mL/min		45 min	UV 360 nm	[54]		
Quercetin, rutin and isorhamnetin		Soxhlet extraction with methanol	BDS-C18 (Agilent, 4.6 mm × 250 mm, 5 μm)	Methanol-0.2% phosphoric acid (52:48); flow rate: 1.0 mL/min		16 min	UV 260 nm	[8]		
Quercetin, rutin and isorhamnetin		Ultrasonic extraction with 60% (v/v) ethanol aqueous solution	C18 (Agilent, 4.6 mm × 250 mm, 5 μm)	Methanol-0.2% phosphoric acid (54:46); flow rate: 1.0 mL/min		15 min	UV 261 nm	[55]		
Quercetin, quercitrin, isoquercitrin and quercetin-7-O-α-l-rhamnoside	Refluxing extraction with 60% (v/v) ethanol aqueous solution		SB-C18 (Agilent ZORBAX, 4.6 mm × 250 mm, 5 μm)	Acetonitrile-0.5% acetic acid (12:88) with gradient elution; flow rate: 1.0 mL/min		45 min	UV 360 nm	[56]		
Quercetin, quercitrin, isoquercitrin and quercetin-7-O-α-l-rhamnoside	Refluxing extraction with water		HC C18 (Agilent, 4.0 mm × 250 mm, 5 μm)	Methanol-2.5% acetic acid (36:64); flow rate: 1.0 mL/min		50 min	UV 255 nm	[57]		
Quercetin, quercitrin, isoquercitrin, taxifolin-7-O-α-l-rhamnoside and kaempferol	Ultrasonic extraction with 50% (v/v) methanol aqueous solution		C18 (Luna, 4.6 mm × 250 mm, 5 μm)	Methanol-0.5% acetic acid (54:46); flow rate: 1.0 mL/min		50 min	UV 350 nm	[58]		
Quercetin, quercitrin, isoquercitrin, quercetin-7-O-α-l-rhamnoside and taxfolin-7-O-α-l-rhamnoside	Ultrasonic extraction with 70% (v/v) methanol aqueous solution		SB-C18 (Agilent ZORBAX, 4.6 mm × 250 mm, 5 μm)	Acetonitrile-0.5% formic acid (12:88) with gradient elution; flow rate: 1.0 mL/min		70 min	UV 256 nm and MS	[59]		
Quercetin, quercitrin, isoquercitrin, rutin, kaempferol and quercetin-3-O-galactoside	Refluxing extraction with 80% (v/v) methanol aqueous solution		C18 (Alltima, 4.6 mm × 250 mm, 5 μm)	Acetonitrile-0.8% acetic acid (11:89) with gradient elution; flow rate: 0.8 mL/min		70 min	UV 254 nm and MS	[60]		
Quercetin, quercitrin, isoquercitrin, quercetin-7-O-α-rhamnoside, 3,4-dihydroxybenzoic acid, taxifolin-7-O-α-l-rhamnoside, 5,7-dihydroxy-2-isopropyl and chormone-8-β-D-glucoside	Ultrasonic extraction with 70% (v/v) methanol aqueous solution		XB-C18 (Ultimate, 4.6 mm × 250 mm, 5 μm)	Methanol-water (5:95) with gradient elution; flow rate: 1.0 mL/min		100 min	UV 254 nm and MS	[61]		

## 5. Pharmacokinetics

A HPLC-DAD method was established and applied successfully to the pharmacokinetic study of quercitrin and isoquercitrin in rat plasma after oral administration of 80% ethanol extract of *H. japonicum* at a dose of 23.0 g/kg, equivalent to 48.3 and 62.1 mg/kg of quercitrin and isoquercitrin, respectively. The pharmacokinetic results suggested that the maximum concentrations of quercitrin and isoquercitrin occured after approximately 1.30 and 1.17 h, respectively. Quercitrin and isoquercitrin were still detectable in rat plasma for at least 24 h after oral administration of the extract. These findings provided useful evidence for evaluating the clinical efficacy of *H. japonicum* [68].

## 6. Conclusions

Pharmacological studies on flavonoids have been performed *in vitro* and *in vivo* in animals, while the pharmacological studies on other main bioactive components such as phloroglucinols and xanthones are rare. Though several pharmacological mechanisms related to biological activity have already been explained, the comprehensive pharmacological mechanisms of *H. japonicum* need to be elucidated. Based on phytochemical and pharmacological research, the flavonoids responsible for the good hepatoprotective, anti-tumor and antibacterial activities were selected as chemical markers to evaluate the quality of *H. japonicum* and its products. Meanwhile, various methods have been successfully applied to the simultaneous analysis of the bioactive compounds in *H. japonicum*. However, pharmacokinetics studies on the main components, especially the bioactive components are still largely lacking, therefore firm evidence for further clinical application is necessary in order to assess the therapeutic potential of *H. japonicum* and its pharmaceutical commodities.
